# Immunisation Rates of Medical Students at a Tropical Queensland University

**DOI:** 10.3390/tropicalmed3020052

**Published:** 2018-05-23

**Authors:** Erin Fergus, Richard Speare, Clare Heal

**Affiliations:** 1 School of Medicine and Dentistry, James Cook University, Mackay 4740, Australia; clare.heal@jcu.edu.au; 2 Anton Brent Centre for Health System Strengthening, James Cook University, Townsville 4811, Australia

**Keywords:** medical students, healthcare students, immunisation, vaccination, occupational diseases, infection control

## Abstract

Although medical students are at risk of contracting and transmitting communicable diseases, previous studies have demonstrated sub-optimal medical student immunity. The objective of this research was to determine the documented immunity of medical students at James Cook University to important vaccine-preventable diseases. An anonymous online survey was administered thrice in 2014, using questions with categories of immunity to determine documented evidence of immunity, as well as closed-ended questions about attitudes towards the importance of vaccination. Of the 1158 medical students targeted via survey, 289 responses were included in the study (response rate 25%), of which 19 (6.6%) had documented evidence of immunity to all of the vaccine-preventable diseases surveyed. Proof of immunity was 38.4% for seasonal influenza, 47.1% for pertussis, 52.2% for measles, 38.8% for varicella, 43.7% for hepatitis A, and 95.1% for hepatitis B (the only mandatory vaccination for this population). The vast majority of students agreed on the importance of vaccination for personal protection (98.3%) and patient protection (95.9%). In conclusion, medical students have sub-optimal evidence of immunity to important vaccine-preventable diseases. Student attitudes regarding the importance of occupational vaccination are inconsistent with their level of immunity. The findings of this study were used to prompt health service and educational providers to consider their duty of care to manage the serious risks posed by occupational communicable diseases.

## 1. Introduction

Immunisation of medical students is an important infection control strategy, one that is strongly recommended by leading international public health advisory bodies [1,2]. Clinical guidelines for vaccination decision-making in Australia have been developed by the Australian Technical Advisory Group on Immunisation. Occupational vaccination recommendations from this group state that healthcare workers and students should ensure immunity to hepatitis B, seasonal influenza, measles, mumps, rubella, pertussis, and varicella. Additionally, those who work in remote Indigenous communities or with Indigenous children should be vaccinated against hepatitis A [3]. Adherence to these recommendations is mandated variably across Australian health services and universities—there is no national legislated requirement for occupational vaccination. For Australian-born medical students, many of these vaccinations would have been provided through a government-subsidised childhood immunisation scheme. However, for adults who are not in high-risk medical populations, any additional vaccines are the financial responsibility of the individual [3]. Private health insurance providers in Australia are not required to reimburse for vaccine-related expenses. 

Despite the strength of occupational vaccination recommendations, medical students consistently have sub-optimal immunity to vaccine-preventable diseases, as was highlighted in a recent review of the literature on vaccine coverage among healthcare students [4]. The only published Australian research on medical student immunity was undertaken between 2002 and 2005 at the University of New South Wales. Using questionnaires and serological testing, the authors concluded that a significant proportion of first-year medical students were not immune to important vaccine-preventable diseases [5]. 

The primary objective of this study was to determine the documented immunity of medical students at a tropical Queensland university to important vaccine-preventable diseases. The findings were used to inform health service and educational providers about the adequacy of their current immunisation policies. 

## 2. Organisational Context

Medical students at James Cook University, Queensland, Australia, are enrolled in a six-year undergraduate degree. Clinical exposure commences in first year and increases proportionally with progress through the course. Students in years one, two, and three are considered ‘pre-clinical’, receiving most of their education (including patient interaction) within the university environment. Students in years four, five, and six are in their ‘clinical’ years of medical school; the majority of their teaching takes place in hospitals. The main medicine campus is in Townsville, with other centres located across Northern Australia in Cairns, Mackay, and Darwin. Medical students are financially responsible for their immunisation-related expenses. They are sometimes included in Queensland Health staff vaccination initiatives, but not in all facilities. As per James Cook University and Queensland Health policy at the time of this research in 2014, healthcare students were required to provide proof of seroconversion to hepatitis B. The remainder of the immunisation schedule was recommended but not mandatory. These policies have since been updated [6,7].

## 3. Materials and Methods 

**Figure 1:**
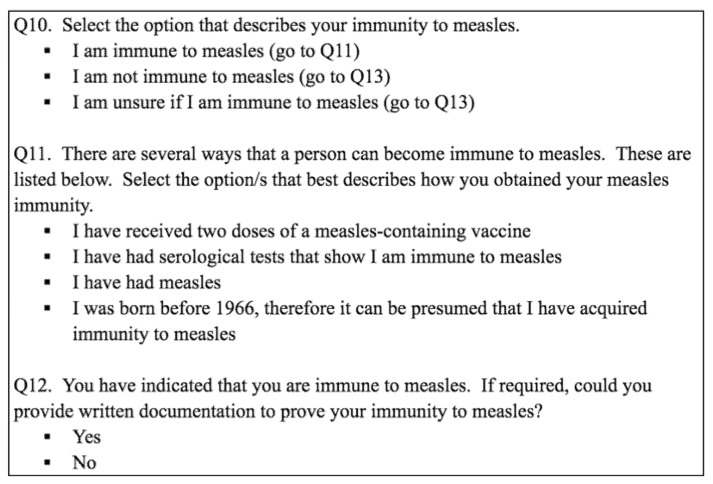
Use of categories to define measles immunity.

An anonymous online survey was administered to medical students at James Cook University. The questions in the survey were specifically designed to ascertain history of documented immunity to important vaccine-preventable diseases (influenza, pertussis, measles, varicella, hepatitis A, and hepatitis B). These diseases were selected based on their significant potential for nosocomial transmission in this medical student population. Categories were used to define immunisation status, using proof of immunity guidelines from the Australian Immunisation Handbook and the Centers for Disease Control and Prevention [1,3]. Figure 1 demonstrates the use of this category system. Included in the survey were questions about socio-demographic variables. There were also two closed-ended questions about student attitudes towards the importance of occupational vaccination.

The survey was piloted on a group of ten final-year medical students. Emails were sent to medical students enrolled in all six years on three occasions during July and August 2014. A hyperlink directed students to the information statement and informed consent document, followed by the survey. The hyperlink was also posted on social media and promoted by the James Cook University Medical Students Association. This study was approved by the Human Research Ethics Committee at James Cook University (approval number H5664). 

Data was collected by SurveyMonkey (www.surveymonkey.com) and analysed using SPSS for Windows, version 22.0 (IBM, New York, NY, USA). Incomplete responses were removed from the data set prior to analysis. Students who were ‘unsure’ of their vaccination status were grouped with the unvaccinated students for further analysis. Those who were unable to seroconvert to hepatitis B were considered immune, given that in the years after vaccination up to 60% of people lose detectable antibody but not protection [8]. Data were rigorously examined for error. Descriptive analyses were employed. Pearson’s chi-square tests were used to investigate for statistically significant relationships between immune status and the independent variables (age, gender, nationality, year level group, and campus). Frequency tables were used to determine completeness of student vaccine coverage. Pearson’s chi-square tests were used again to investigate for significant associations between completeness of vaccination and the independent variables. 

## 4. Results

Of 1158 enrolled medical students, 289 students (25%) across the four James Cook University medicine campuses completed the survey (33 surveys that were only partially completed were not included). The majority of students were aged between 18 and 24 (86.5%), were female (68.9%), and had grown up in Australia (82.7%). When compared to the demographic profile of the James Cook University medical student population in 2014, the sample is well matched in terms of year level group distribution; however, females are over-represented in the sample population (Table 1). 

The mandatory hepatitis B vaccine had the highest rate of documented immunity at 95%, while measles was 52.2% and all other vaccines surveyed were less than 50% (Table 2 and Table 3). There was a statistically significant association between influenza immunity and medical student seniority—54.7% of clinical students received the influenza vaccine in 2014, compared to 23.3% of pre-clinical students (*p* < 0.001). Pre-clinical or clinical year level group did not predict immunity to pertussis, measles, varicella, hepatitis A, or hepatitis B (Table 4). There were no statistically significant associations between immunity to any of the diseases and student age, gender, campus, or nationality (*p* > 0.05). Notably, the majority of students perceived vaccination as important for their personal protection (11.1% agree, 87.2% strongly agree); as well as for patient protection (11.8% agree, 84.1% strongly agree). 

The proportion of students with documented immunity to all of the diseases surveyed was 6.6% (19/289). The remaining 93.4% of respondents would fulfil criteria for one or more catch-up immunisations. Administration of 823 vaccination catch-up schedules for individual diseases would be recommended to the students surveyed: an average of 3.05 per survey respondent. There were no statistically significant associations between comprehensiveness of vaccine coverage and year level group, age, gender, campus, or nationality (*p* > 0.05). 

## 5. Discussion

The majority of medical students (93.4%) in this study were assessed as needing at least one vaccine. This suggests that there is significant vulnerability to communicable disease among this population, with resultant public health implications for hospital staff and patients and the university community. This population’s strong belief in the importance of occupational vaccination is inconsistent with their low levels of immunity, suggesting that there is a need for research into other factors that influence medical student vaccination uptake. 

Catch-up immunisations were recommended for 74% of medical students in a paediatric hospital in Basel, Switzerland [9], which is comparable to the findings of this study. Similarly, less than 30% of medical and nursing students in an Athenian study were in full compliance with recommended vaccinations [10]. In this study, documented immunity to recommended vaccines was lower than that demonstrated in Lille, France—72.7% of the French medical students had proof of immunity to pertussis, 78% had proof of immunity to measles, and 78.9% had proof of immunity to varicella [11]. Hepatitis B immunity was documented in 91.8% of French healthcare students, which is similar to our findings [12]. Among medical students studying at James Cook University, there was no statistically significant difference in immunity between those who grew up in Australia and those who grew up in other parts of the world. There was also no difference between the medical school campuses. These negative findings serve to reiterate that sub-optimal medical student immunity is not limited by geographic boundaries. 

The rates of influenza vaccine uptake in this study were higher than the rates observed among medical students in Strasbourg, Warsaw, and Teheran (29.7%, 15.2%, and 4.7%, respectively) [13]. Sub-optimal influenza vaccination in other healthcare worker populations sets a poor example for medical students. A review of the literature pertaining to seasonal influenza vaccination among Australian hospital healthcare workers found that rates ranged from 16.3% to 58.7% (29% to 53% for physicians) [14]. The majority of studies into healthcare worker immunity have focused on seasonal influenza, but there is research that has demonstrated poor Australian healthcare worker compliance with recommended vaccination schedules [15]. These findings suggest that doctors may be poor vaccination role models for medical students. 

In this study, medical students in their clinical years were more likely to be vaccinated against seasonal influenza than their more junior pre-clinical colleagues. There are several potential explanations for this finding. Knowledge, specifically regarding disease severity and vaccine safety, has previously been identified as an important determinant of medical student immunization behaviour [13,16,17]. Higher rates of influenza immunity in more senior students could therefore be attributed to acquisition of knowledge during medical school. However, year level was not associated with increased immunity to any of the other diseases surveyed. This could suggest a difference in the way that influenza teaching is delivered. Alternatively, it is possible that clinical medical students are more often opportunistically included in seasonal staff vaccination clinics during their hospital and community placements. 

The levels of documented hepatitis B immunity among North Queensland medical students are high, which is attributable to the mandatory government and university requirement to be immune to this disease. Interestingly, documented hepatitis A immunity was similar to the other diseases surveyed, despite it not being included in the Australian childhood vaccination schedule. Hepatitis A vaccination is routinely recommended to travellers, thus the holiday patterns of medical students may be impacting their vaccination behaviour (other potential influences, although admittedly less likely, include the desire to safely consume raw oysters and semi-dried tomatoes [18,19]). Another explanation is that North Queensland medical students have responded to the recommendation that all healthcare workers and students practising in Indigenous Australian communities have immunity to hepatitis A. Nevertheless, this seems less likely, given the generally poor uptake of the non-mandatory vaccinations in this population. 

The rate of self-reported immunity to pertussis was higher in this study than in the general Australian adult population. In the 2009 Adult Vaccination Survey, conducted by the Australian Institute of Health and Welfare, 11.3% of respondents reported being vaccinated against pertussis as an adult or adolescent. The Adult Vaccination Survey also noted that only 18.9% of adult Australians received the free pandemic (H1N1) influenza vaccine in 2009 [20]. Rates of seasonal influenza uptake in this medical student population were similar to those reported in Australian adults in 2014 (39%); however, the students fared more favourably when compared to younger Australian adults (24% influenza vaccine uptake in those aged 18–24 years; 23% in those aged 25–34 years) [21]. 

The first limitation of this study is the low survey response rate (25%), although the year level distribution of the sample population is well matched to the known demographic characteristics of the James Cook University medical student population. A second limitation of this study is its reliance on self-reporting of immunisation status (due to resource and funding constraints that precluded collection of serological data). However, it is recognised that the most important requirement for assessment of vaccination status is to have written documentation of vaccination, and for most diseases, there are no adverse events associated with re-vaccination of adults [3]. Thus, the category system specifically requesting documented proof of immunity that was utilised in this study should be considered an acceptable method of confirming vaccination history when serological data is unavailable. 

## 6. Outcomes and Recommendations

This study highlighted the important need to address the vaccination rates of medical students, a population who, theoretically, should be extremely motivated to ensure their immunity to common vaccine-preventable diseases. Shortly following the acquisition of these survey results, qualitative research was undertaken on this medical student population to identify the determinants of their vaccination behaviour. Strategies to improve immunity were identified and published [22]. The vaccination policy for healthcare students at James Cook University has subsequently been updated since these results were provided to the organisation in 2014—prior to clinical exposure, students are now required to provide proof of immunity to measles, mumps, rubella, varicella, and pertussis, in addition to hepatitis B [23]. There has also been a medical student-led influenza vaccination campaign, which received national acclaim in 2017 [24]. Future research efforts could focus on exploring the impact of mandatory vaccination on medical student beliefs and behaviours. 

It is evident that medical students cannot be relied upon to ensure their own immunity. Other health service and educational providers must reflect on their current immunisation policies and take action in order to protect the health of their students and the wider community. 

## Figures and Tables

**Table 1:** Demographic profile of the sample population compared with the James Cook University (JCU) medical student population in 2014.

Student Demographics	Sample Population (*n* = 289)	JCU Medical Students (*n* = 1158)	Difference (%)
Females	199 (68.9%)	669 (57.7%)	11.2%
Pre-clinical students	150 (51.9%)	635 (54.8%)	−2.9%
First year	48 (16.6%)	219 (18.9%)	−2.3%
Second year	47 (16.2%)	220 (19%)	−2.8%
Third year	55 (19%)	196 (16.9%)	2.1%
Clinical students	139 (48.1%)	523 (45.2%)	2.9%
Fourth year	38 (13.1%)	170 (14.7%)	−1.6%
Fifth year	47 (16.3%)	191 (16.5%)	−0.2%
Sixth year	54 (18.7%)	162 (14%)	4.7%

**Table 2:** Rates of self-reported seasonal influenza vaccination among medical students.

Disease	Vaccination Status		
	Vaccinated	Not Vaccinated	Unsure If Vaccinated
Seasonal influenza (2013)	113 (39.1%)	172 (59.5%)	4 (1.4%)
Seasonal influenza (2014)	111 (38.4%)	176 (60.9%)	2 (0.7%)

**Table 3:** Rates of documented immunity to selected vaccine-preventable diseases among medical students.

Disease	Immunisation Status			
	Immune with Proof	Immune without Proof	Not Immune	Unsure of Status
Pertussis	136 (47.1%)	48 (16.6%)	33 (11.4%)	72 (24.9%)
Measles	151 (52.2%)	69 (23.9%)	4 (1.4%)	65 (22.5%)
Varicella	112 (38.8%)	114 (39.4%)	8 (2.8%)	55 (19%)
Hepatitis A	126 (43.7%)	33 (11.4%)	38 (13.1%)	92 (31.8%)
Hepatitis B	275 (95.1%) ^1^	10 (3.5%)	1 (0.3%) ^2^	3 (1%)

^1^ Nine respondents (3.1%) unable to seroconvert; ^2^ one respondent (0.3%) with active hepatitis B infection.

**Table 4:** Rates of documented immunity among year level groups.

Disease	Proportion of Students with Evidence of Immunity		
	Pre-Clinical Medical Students (*n* = 150)	Clinical Medical Students (*n* = 139)	*p*-Value
Influenza	23.3%	54.7%	<0.001
Pertussis	48%	46%	0.739
Measles	48.7%	56.1%	0.205
Varicella	38.7%	38.8%	0.975
Hepatitis A	41.3%	46%	0.420
Hepatitis B	96%	94.2%	0.487
